# Respondents of health survey powered by the innovative NURO app exhibit correlations between exercise frequencies and diet habits, and between stress levels and sleep wellness

**DOI:** 10.3389/fpsyt.2022.945780

**Published:** 2022-09-06

**Authors:** Daniel Gallucci, Ernest C. Y. Ho, Joseph Geraci, Joseph Loren, Luca Pani

**Affiliations:** ^1^Nurosene Health Inc., Toronto, ON, Canada; ^2^Department of Molecular Medicine, Queen's University, Kingston, ON, Canada; ^3^Center for Biotechnology and Genomics Medicine, Medical College of Georgia, Augusta, GA, United States; ^4^Department of Biomedical, Metabolic and Neural Sciences, University of Modena and Reggio Emilia, Modena, Italy; ^5^Department of Psychiatry and Behavioral Sciences, Leonard M. Miller School of Medicine, University of Miami, Miami, FL, United States

**Keywords:** digital health technology, smartphone application, machine learning, mental and physical wellbeing, survey questionnaire, variational auto encoder, exploratory factor analyses, confirmatory factor analyses

## Abstract

Nurosene's NURO app (nurosene.com) is an innovative smartphone application that gathers and analyzes active self-report metrics from users, empowering them with data-driven health machine intelligence. We present the data collected and analyzed from the initial round of participants who responded to a 12-question survey on their life-style and health status. Exploratory results using a variational autoencoder (VAE) suggested that much of the variability of the 12 dimensional data could be accounted for by two approximately uncorrelated latent variables: one pertaining to stress and sleep, and the other pertaining to exercise and diet. Subsequent modeling of the data using exploratory and confirmatory factor analyses (EFAs and CFAs) found that optimal data fits consisted of four factors, namely exercise, diet, stress, and sleep. Covariance values were high between exercise and diet, and between stress and sleep, but much lower between other pairings of non-identical factors. Both EFAs and CFAs provided extra contexts to and quantified the more preliminary VAE observations. Overall, our results significantly reduce the apparent complexity of the response data. This reduction allows for more efficient future stratification and analyses of participants based on simpler latent variables. Our discovery of novel relationships between stress and sleep, and between exercise and diet suggests the possibility of applying predictive analytics in future efforts.

## 1. Introduction

Digital health technologies (DHTs) are defined as systems that use computing platforms, connectivity, software, and sensors for healthcare and related purposes. These technologies span a wide range of uses, from applications in general wellness to applications as a medical device. They include technologies intended for use as a medical product, in a medical product, or as an adjunct to other medical products (devices, drugs, and biologics). They may also be used to develop or study medical products. (1). The advent and recent regulatory guidance (2) of DHTs have revolutionized healthcare across numerous domains. In many situations, DHTs improve accessibility, remote monitoring, precision interventions, preventative strategies, and optimize models of care and performance when compared with more conventional means of health care delivery and monitoring.

A key impetus for the accelerated market uptake of DHT platforms comes from their improved efficiency in collecting users' health-related data from their own personal devices such as smartphones. Many forms of quantitative data that used to be collected *via* lab or clinic visits can now be captured more frequently, conveniently, and passively by various devices' sensors. The ease and comprehensiveness of such modes of data collection vastly improve the functionalities of these platforms, resulting in their increasing popularity worldwide.

Besides quantitative or passive monitoring, incorporating DHT data also means more reliance on active self-reported metrics in lieu of/in addition to lab or clinic visits. Collection of self-report metrics in the form of questionnaires has been used in scientific and behavioral research for decades (3). Digital health companies are utilizing questionnaires in an innovative manner to complement and support health systems, as well as empower users to make better informed decisions that contribute to better health outcomes (4).

A prominent example would be the release of Apple's Research Kit in 2015 which leveraged digital health technology *via* the smartphone to reach clinical trial participants across five distinct domains: asthma, breast cancer, cardiovascular disease, diabetes, and Parkinson's disease. The project revolutionized the world of randomized controlled trials (RCTs) as 70,000 plus patients were screened without ever leaving the comforts of their own home. Questionnaires were a valuable tool used systematically and repeatedly in each of the five trials (5).

Research continues to expand beyond the traditional confines of RCTs in clinical/academic settings into “real-world” evidence data as a way of more effectively evaluating population-level health dynamics (6). In this realm, questionnaires remain a valuable tool to gain specific insights from large groups of people at a relatively low cost. The data gathered is versatile as questions can be designed to learn about individuals as well as large groups across almost any issue. Results can also be extrapolated from smaller groups to a larger population without having to ask questions of every single person (7). Nevertheless, initiatives and projects that take advantage of the established effectiveness of self-report metrics and the penetrating power of digital technologies to reach out to the general population or even a specific demographic are still in relative nascence.

Nurosene Health (nurosene.com) is a digital health company focused on identifying hidden relationships among numerous lifestyle factors to enable precision, proactive health strategies to individuals at scale *via* a proprietary app. To achieve this goal, the app incorporates a collection of active self-report metrics as a means to gauge life-style and health statuses of participants. We currently employ several machine learning and statistical methods to understand the inter-relationships of the data collected. Using data-driven machine intelligence the results are then returned to participants empowering their health conscious choices. The app provides the ability to go beyond traditional “in clinic” models by approximating the complexities of life when looking at the dynamic relationships between health, behavior and human performance. These benefits combined with the ubiquity of smartphones will lead to exciting and expansive opportunities ahead as humans and these types of technology continue to coalesce into the future (8, 9).

## 2. Methods and materials

### 2.1. Survey questionnaire

The survey employed by the NURO app consisted of twelve questions. Each question focused on one of the four types of life-style choices or health statuses about the user: exercise, diet, stress and sleep (Table 1). The survey was the result of an author's (DG) experience on the front line of elite athletic performance and neurological rehabilitation for almost two decades, and was initially used in his clinic as a standardized set of questions for understanding the overall health background of the subjects. The NURO app was marketed *via* social media and direct marketing campaigns during the summer of 2021. In a matter of weeks over 1K survey responses were recorded. Each question was in a multiple-choice format. The response of each question for each user was converted to a numeric score according to the scheme in Table 2 for subsequent data analysis.

**Table 1:** The 12 questions on the questionnaire.

**Q**	**Type**	**Question**
1	Exercise	How much cardiovascular and/or aerobic exercise do you get on a weekly basis? (e.g., running, bike riding, swimming)
2	Exercise	How much anaerobic exercise do you get on a weekly basis? (e.g., weight training, circuit training)
3	Exercise	How often would you consider yourself to be cognitively engaged and/or stimulated?
4	Stress	How often are you experiencing physical stress? (e.g., injury, chronic pain, gut distress)
5	Stress	How often are you experiencing psychological stress? (e.g., loneliness, trauma, conflicts)
6	Stress	How often are you experiencing social stress? (e.g., stress with others and life events in general)
7	Diet	How often are you eating what you consider to be a “healthy” diet?
8	Diet	How often do you consume nutritional supplements and/or compounds?
9	Diet	How often do you experience gastrointestinal issues after eating? (e.g., bloating, gas, indigestion)
10	Sleep	How often do you have difficulty falling asleep?
11	Sleep	How often do you have difficulty staying asleep?
12	Sleep	How often do you have difficulty waking up in the morning?

**Table 2:** Scoring scheme.

**Q**	**4**	**3**	**2**	**1**
1	5+ days	3–4 days	1–2 days	None
2	5+ days	3–4 days	1–2 days	None
3	Always	Often	Occasionally	Rarely/never
4	Rarely/Never	Occasionally	Often	Always
5	Rarely/Never	Occasionally	Often	Always
6	Rarely/Never	Occasionally	Often	Always
7	Daily	Often	Occasionally	Rarely/never
8	Daily	Often	Occasionally	Rarely/never
9	Rarely/Never	Occasionally	Often	Daily
10	Rarely/Never	Occasionally	Often	Daily
11	Rarely/Never	Occasionally	Often	Daily
12	Rarely/Never	Occasionally	Often	Daily

Except the first column, the name of each column (“4”, “3”, “2”, “1”) denotes the numeric score used for each question for analyses. Each entry of a respective column is the actual response choice of the question corresponding to the converted numeric score. The scheme is so designed that a higher score indicates superior performance of respondents on the particular survey question.

### 2.2. Variational autoencoder (VAE)

**Figure 1:**
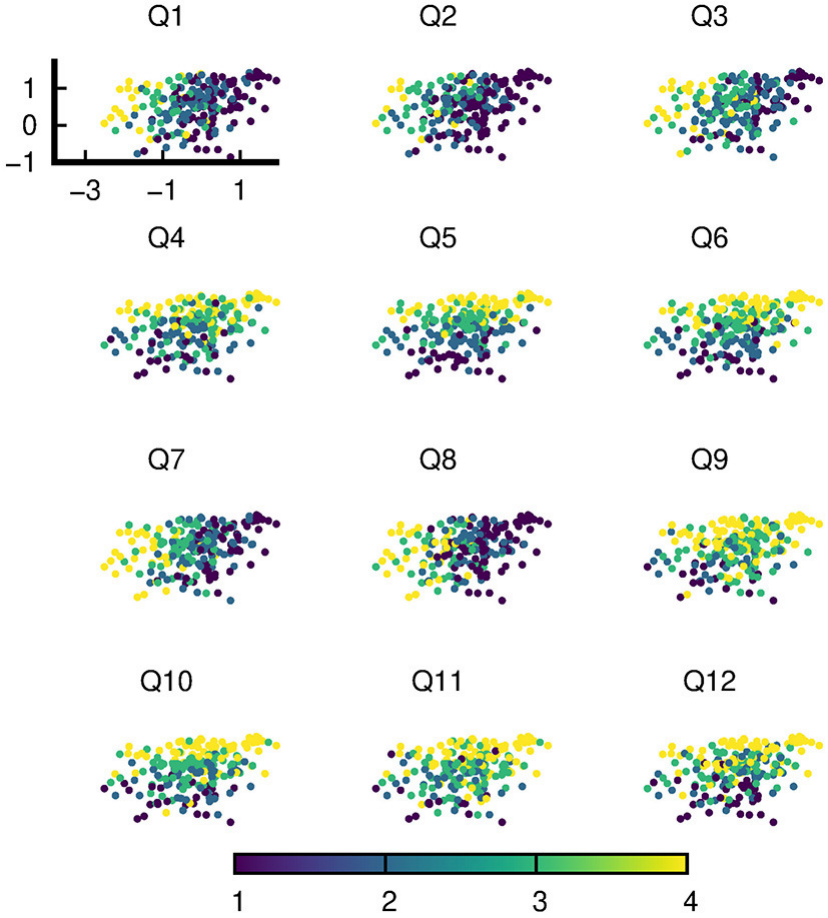
An instance of latent variables of a random subset of data on the trained VAE. The VAE was trained with this very subset of data. Each of the 12 sub-figures is color coded with the responses of one of 12 questions (Q1-Q12).

The VAE was written using Keras [version 2.3.0, (10)], while the training was performed with TensorFlow 2.0.1 (11) on Python 3.7.10 (12). There was one layer for encoding and two layers for decoding in the utilized VAE. The encoder consisted of a dense layer. This dense layer took a (*b*_*size*, 12) tensor as input, and outputted a (*b*_*size*, 6) tensor (*b*_*size* is the batch size used for VAE training). The “reparametrization” layer took the output from the encoder layer as input and mapped it to the 2D latent or code layer. The decoder had two dense layers. The first layer took the latent variables as input and outputted a (*b*_*size*, 6) tensor, which was then subsequently fed into the other dense layer and transformed to a tensor identical in dimensions to the original input of the response data (*b*_*size*, 12). The scatter plot of the subset of data used for training on the VAE (Figure 1) was generated by gnuplot [version 5.2.8, (13)] with the viridis color palette (14).

### 2.3. Exploratory and confirmatory factor analyses (EFAs and CFAs)

**Figure 2:**
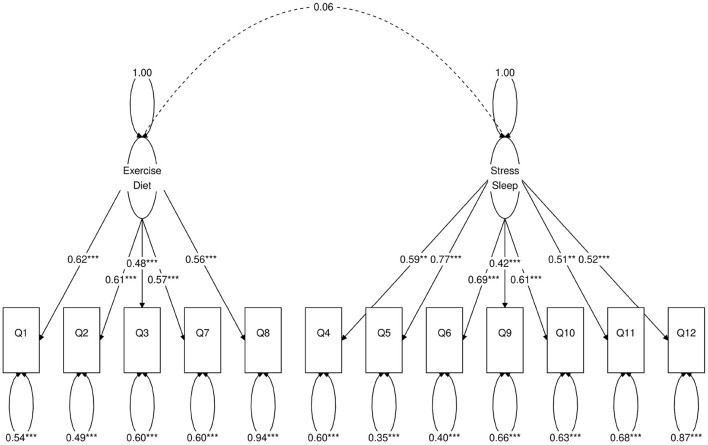
Path diagram of the two-factor CFA model. *Besides a value means statistical significance. 1-star denotes *p* < 0.05, 2-star denotes *p* < 0.01, 3-star denotes *p* < 0.001.

**Figure 3:**
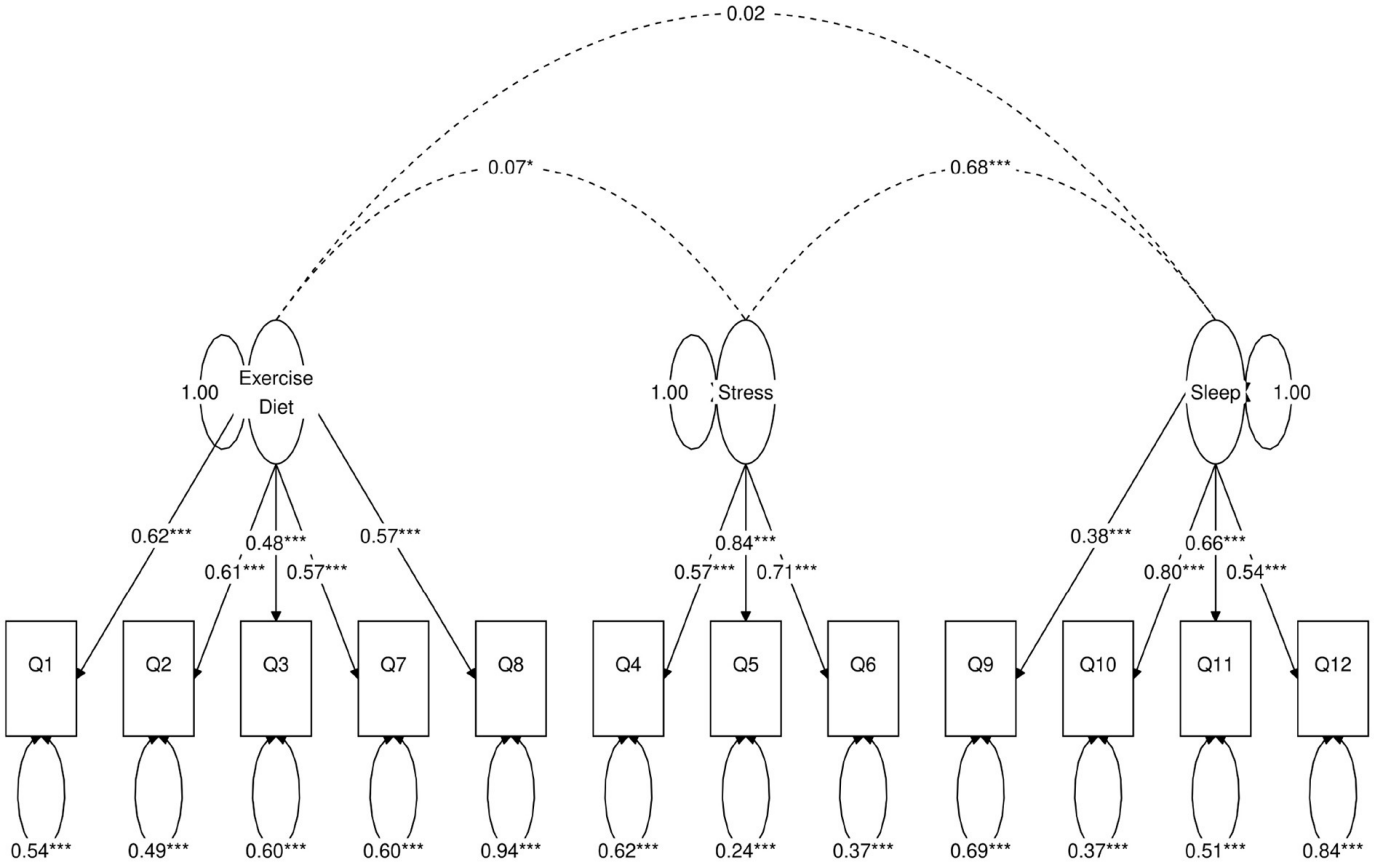
Path diagram of the three-factor CFA model. Star conventions as in Figure 2.

**Figure 4:**
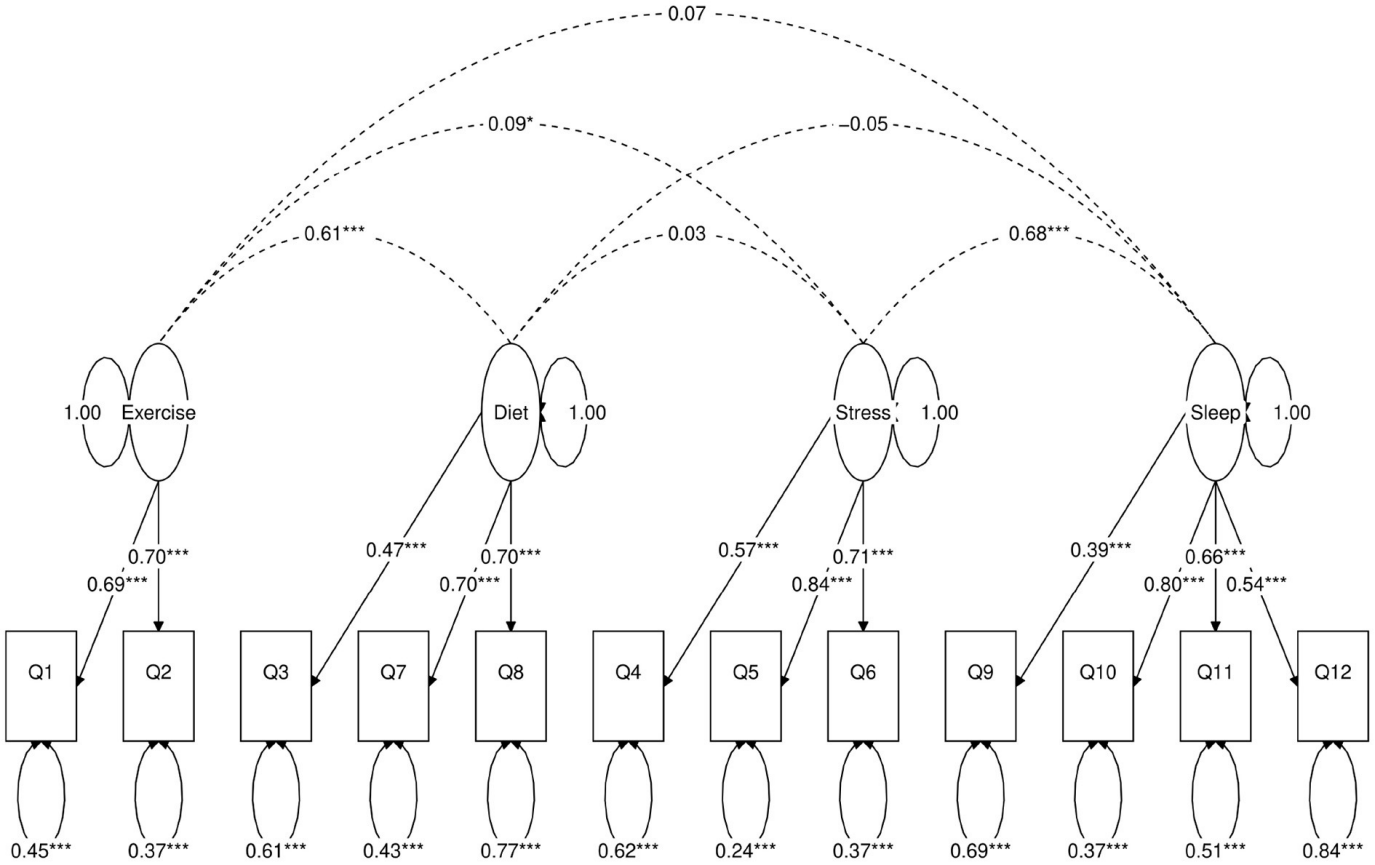
Path diagram of the first four-factor CFA model (Model A). Star conventions as in Figure 2.

**Figure 5:**
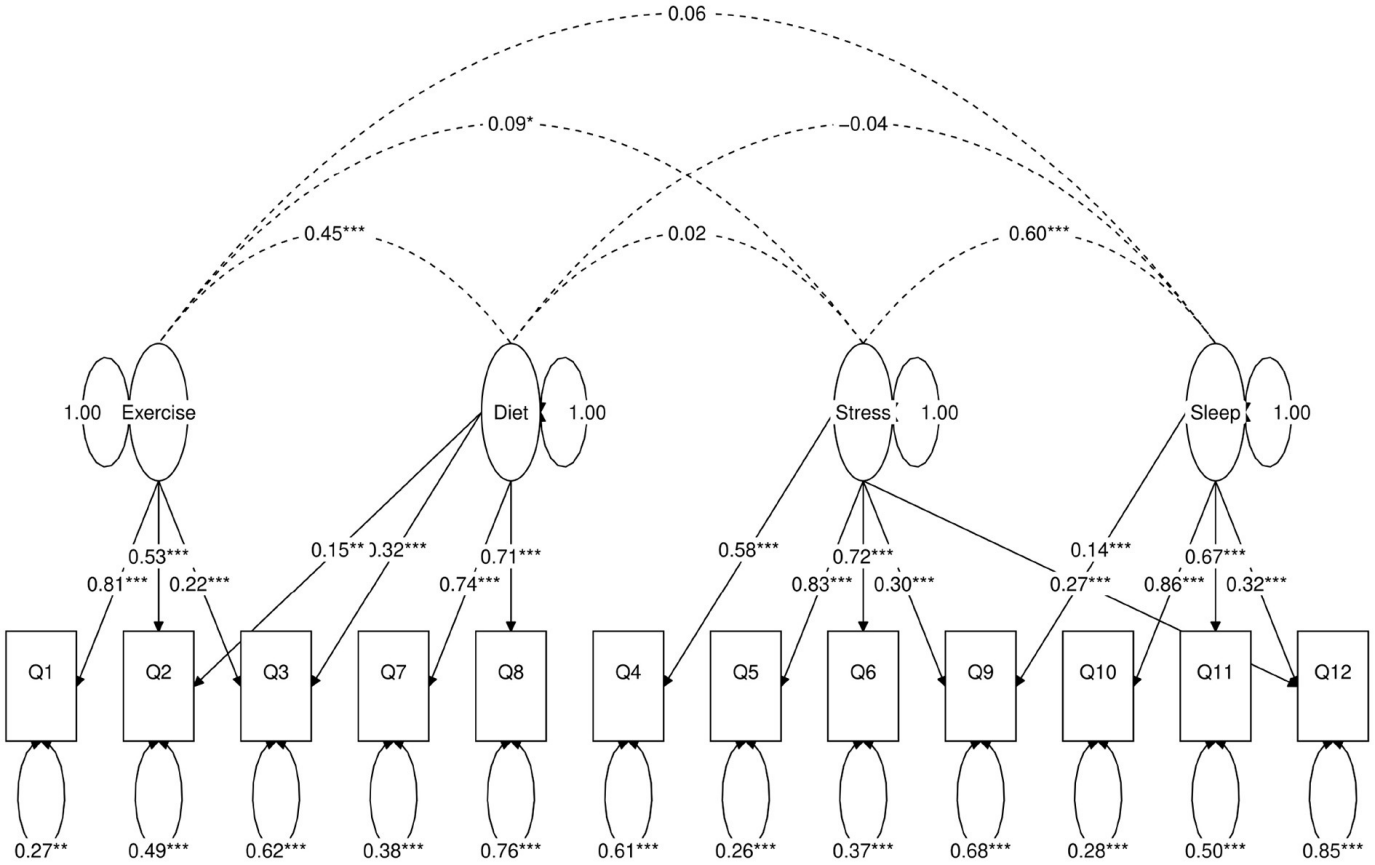
Path diagram of the second four-factor CFA model (Model B). Star conventions as in Figure 2.

The fa function of the psych package [version 2.2.5, (15)] was used to perform exploratory factor analyses. We chose the default minres (“minimal residual”) as the factoring method in the fm option and the default oblique oblimin as the factor rotation method in the rotate option of the function. The cfa function of the lavaan package [version 0.6-11, (16)] was used for performing CFAs on the response data. The optimization of CFA model parameters was based on the default maximal likelihood approach (estimator=“ML”) of the cfa function. In no CFA model was there any evidence of irregularity in the optimization procedure, as each procedure exited normally within the default number of maximum iterations. The path diagrams (Figures 2–**5**) were constructed using the tidySEM package [version 0.2.3, (17)]. The skewness and kurtosis quantities of the responses for each question item (Table 3) were obtained using the moments package [version 0.14.1, (18)]. Every package in this section was R based. All work in this section was performed on version 4.0.5 of R software (19).

**Table 3:** Basic statistical quantities of the responses for each question item.

**Statistical quantity**	**Q1**	**Q2**	**Q3**	**Q4**	**Q5**	**Q6**	**Q7**	**Q8**	**Q9**	**Q10**	**Q11**	**Q12**
Mean	1.95	1.76	2.26	2.81	2.66	2.78	2.28	2.17	3.06	2.87	3.02	2.87
SD	0.96	0.93	0.91	0.97	0.97	0.94	0.96	1.12	0.92	1.00	0.97	1.07
Skewness	0.71	0.96	0.26	−0.32	−0.16	−0.35	0.25	0.46	−0.69	−0.43	−0.60	−0.45
Kurtosis	2.49	2.80	2.25	2.08	2.02	2.24	2.10	1.82	2.59	2.07	2.27	1.92

SD stands for standard deviation.

## 3. Results

In this work, we utilized data from an early group of NURO app users who responded to our intake survey (*N* = 1315) during the summer of 2021 (Supplementary File 1). The survey consisted of twelve life-style and wellbeing related questions (Table 1). Participants could choose from one of the four possible responses for each question (Table 2) in multiple choice format. These questions originated from the elite athletic performance and neurological rehabilitation clinic led by one of the authors (DG), and were designed based on two emerging principles in the field of rehabilitation and performance optimization: (1) behavioral effects can be related to biological processes (20), and (2) social and behavioral interventions, such as regular physical activity and social support, have positive benefits on brain and body health (21).

Therefore, the questions sought to gather data involving the contextual complexities of human behavior, e.g., two persons may present with identical neurological conditions but may have arrived there *via* entirely different circumstances. The language utilized was kept simple to ensure consistent interpretability and translation to data. The ability to identify and then plot stress over time at the individual level (22) was a fundamental component of our questionnaire in the clinic. What research clearly demonstrated was indeed being seen in clinic: stress was either causative, or a contributor to ailments (23). It also became clear that lifestyle factors revolving around diet, exercise and sleep also needed to be factored in when aiming to determine someone's health status as well as the ability to provide tailored interventions.

The primary reason for including the questionnaire (Table 1) as part of the NURO app was to replicate clinical findings to a larger segment of population, with the eventual goal of promoting individualized physical and mental wellbeing at scale. Upon completion of the survey, the response of each question for each participant was converted to a numeric score from 1 to 4 according to the scheme in Table 2. Thus, the responses of each participant was represented by a numeric (row) vector of 12 elements, with each element being 1, 2, 3, or 4. Information on age and gender were not required nor recorded for this initial round of participants. Table 3 shows the basic statistical quantities of the responses for each of the 12 question items.

The sections below detail the results of our initial exploration of the latent structures of the data using VAE, and the subsequent confirmatory step of extracting the relevant factors and their inter-relationships. These factors were implicated in the VAE exploratory step and were hypothesized to be the low dimensional latent variables that drive the response data.

### 3.1. VAE trained with a random subset of response results reveals low dimensional structures of data

We trained a VAE with a random subset of the response data for initial exploration of potential low dimensional structures. A VAE is a form of artificial neural network that is commonly used to obtain generative probability models of data (24). Sandwiched between the input and output layers of a VAE is a single layer called the code layer or latent space layer. The latent space contains the latent variables conditioned on the input data. The latent space is usually of a lower dimension (in our case two dimensional) than the original data. Thus, examining the structure of the latent space provides an efficient avenue for understanding the input data.

Figure 1 shows an instantiation of the latent variables of the random subset of training data (*N* = 225) on the trained VAE. Each sub-figure is color coded with the responses of a single question. Visual inspection of the distribution of responses over the latent space already clearly shows that despite the higher dimensionality of data (12 responses for 12 questions), the structure of the data can be understood with a lower dimensional manifold.

In particular, the distribution of the responses of each question over the latent space aligns with one of the two roughly uncorrelated axes–the “Diet-Exercise” axis for Q1, Q2, Q3, Q7, and Q8 and the “Stress-Sleep” axis for Q4, Q5, Q6, Q9, Q10, Q11, and Q12. The naming convention of axes does not fully categorize the type of responses with which there is an alignment, for example Q9 is a diet question but aligns with other stress and sleep related questions. Nevertheless, with the exception of Q9, the axes literally paint a picture of the positive correlations between diet and exercise on the one hand, and between stress and sleep on the other.

### 3.2. Exploratory factor Analyses (EFAs) on the same subset of data used for VAE training

To further determine the possible configurations of factors and also to provide additional contexts on the VAE results, we performed exploratory factor analyses (EFAs) on the same subset of data that was used for prior VAE training. In EFAs, all items (the 12 survey question responses) were initially assumed to load on all factors. The purpose of EFA is to estimate the values of these factor loadings, so that the forms of measurement models with simpler structures (for example, models in which each item loads on one factor) can be determined for CFAs.

Table 4 shows the results of the estimated factor loadings of the two-factor, three-factor and four-factor models based on EFAs on the subset of data (*N* = 225). Bold values represent the largest factor loading in absolute magnitude of the item represented by the row in each EFA model.

**Table 4:** Results of exploratory factor analysis on the same randomly selected subset of data used for VAE.

**Q**	**Two-factor**		**Three-factor**			**Four-factor**			
	**F1**	**F2**	**F1**	**F2**	**F3**	**F1**	**F2**	**F3**	**F4**
	**Stress-Sleep**	**Exercise-Diet**	**Exercise-Diet**	**Stress**	**Sleep**	**Stress**	**Sleep**	**Diet**	**Exercise**
1	0.04	**0.67**	**0.69**	−0.07	0.11	−0.03	−0.01	0.01	**1.01**
2	0.11	**0.67**	**0.67**	0.07	0.04	0.14	−0.03	0.30	**0.42**
3	−0.02	**0.55**	**0.54**	0.09	−0.12	0.12	−0.14	**0.34**	0.25
4	**0.66**	0.03	0.02	**0.50**	0.22	**0.53**	0.19	−0.02	0.02
5	**0.73**	0.04	0.00	**0.89**	0.04	**0.88**	−0.04	−0.00	−0.02
6	**0.70**	0.02	−0.01	**0.76**	0.03	**0.77**	0.02	−0.02	−0.02
7	−0.08	**0.68**	**0.68**	−0.05	− 0.05	−0.03	0.05	**0.87**	−0.01
8	−0.14	**0.49**	**0.47**	0.01	− 0.18	0.03	−0.13	**0.53**	0.01
9	**0.57**	-0.09	−0.07	0.30	**0.35**	0.31	**0.33**	−0.06	−0.03
10	**0.63**	−0.04	0.02	− 0.01	**0.82**	−0.01	**0.88**	0.05	−0.02
11	**0.50**	−0.12	−0.08	0.01	**0.60**	0.05	**0.54**	−0.16	0.07
12	**0.59**	0.06	0.08	0.25	**0.42**	0.28	**0.39**	0.00	0.08
Var. exp.	23%	16%	16%	15%	12%	16%	12%	11%	11%
BIC	−83		−104			−92			
TLI	0.77		0.88			0.95			
RMSEA	0.105		0.075			0.051			

Var. exp. denotes the percentage of variance explained by individual factors. TLI stands for Tucker-Lewis index. RMSEA is root mean square error of approximation. BIC stands for Schwarz-Bayes Information Criterion (25). Bold values indicate the largest factor loading in absolute magnitude of the item represented by the row in each EFA model.

It is clear from results of the two-factor EFA model that the composition of factor loadings is in direct agreement with the VAE observation. (Q1, Q2, Q3, Q7, and Q8 align with one factor, while the rest of questions align with the other factor.) In the three-factor EFA model, the extra factor comes from the separation of the original “Stress-Sleep” factor of the two-factor EFA model into two, with one of the new factors being dominated by questions pertaining sleep (Q10, Q11, and Q12), and the other one dominated by questions concerning stress (Q4, Q5, and Q6). In the four-factor EFA model, we further see the original “Exercise-Diet” factor being broken into one factor primarily dominated by diet questions (Q7 and Q8) and one exercise question (Q3), and the other new factor dominated by questions on exercise (Q1 and Q2).

### 3.3. Confirmatory factor Analyses (CFAs) on the entire set of data based on VAE and EFA results

Guided by the intuitions gathered *via* the results of the VAE (Figure 1), we hypothesized that the response data was driven by a relatively small number of factors which are represented as patterns of response distributions over the latent space of the VAE. These small number of factors can also exhibit correlations with one another. In our case for example, a factor that represents “stress” would show a high positive correlation with the factor that represents “sleep”, but, notably, limited correlation with any factor that represents “diet” or “exercise”. The estimated factor loadings from EFAs are also in broad agreement with observations from the trained VAE.

Next, we use a statistical technique called confirmatory factor analysis (CFA) to examine whether the data set (*N* = 1315) fits any model in accordance with our null hypothesis (26–28). CFA is widely used in social and psychiatric research projects [for example, (29–32)]. In our case, we developed a two-factor (Figure 2), a three-factor (Figure 3), and two four-factor (Figure 4) CFA models based on observations of the VAE and EFA results. The fitted CFA model parameters including their statistical significance level are shown in the path diagrams. The manner in which each item loaded on factors in every CFA model followed the results of EFAs (Table 4). In the two-factor (Figure 2) and three-factor (Figure 3) models, each item loaded on one factor as determined by the absolute magnitude of the EFA factor loadings for that item. We maintained this simple structure (each item loads on one factor) and factor assignment scheme (factor with the loading having the largest absolute magnitude for the item concerned) in one of our four-factor CFA models (Model A; Figure 4). However, since “spillings” of loadings were observed in Q2, Q3, Q9, and Q12 in the four-factor EFA model (Table 4), we have as well performed another four-factor CFA model (Model B; Figure 5) in which the above items (Q2, Q3, Q9, and Q12) cross-loaded.

In each of the four CFA model fits, we used the variance standardization method to fix the variance of each factor to unity, with each factor loading and each item variance a free parameter to be fitted. The goodness-of-fit indices of each CFA model are shown in Table 5. Various guidelines have been proposed to interpret these indices for indications of a good model fit. In general, a CFA model with a CFI > 0.90, RMSEA < 0.08, and SRMR < 0.08 (34) is considered a reasonable fit to data. More recently, (35) suggested a more stringent cutoff of CFI at around 0.95, RMSEA at around 0.06 but keeping SRMR at around 0.08. Based on the above criteria, the two-factor model fit (Figure 2) is deemed marginal, while both the three-factor (Figure 3) and four-factor (Figures 4, 5) fits can be regarded as reasonable or good. It is also important to point out that the improvements in many goodness-of-fit indices (such as AIC, BIC, and CFI) drop as the models approach the sophistication of four-factor models. It is an indication that the “optimal” model which balances parsimony and reduction of fitting error should resemble one or both of these two four-factor models. Despite the smaller improvement in AIC and BIC of the four-factor Model B (Figure 5) over Model A (Figure 4) than over other CFA models developed here, the magnitude of AIC or BIC improvement (36) merits our preference of Model B to Model A as the “best” CFA model of the four.

**Table 5:** Summary of CFA model fitting statistics.

**Model**	**DoF**	**AIC**	**BIC**	**CFI**	**RMSEA (90% CI)**	**SRMR**	**Total variance explained**
Two-factor (Figure 2)	53	40,671	40,800	0.83	0.098 (0.092, 0.105)	0.061	36%
Three-factor (Figure 3)	51	40,431	40,571	0.89	0.080 (0.074, 0.087)	0.054	41%
Four-factor A (Figure 4)	48	40,252	40,408	0.94	0.063 (0.056, 0.070)	0.047	45%
Four-factor B (Figure 5)	44	40,133	40,309	0.97	0.047 (0.039, 0.054)	0.034	47%

DoF stands for degrees of freedom, AIC (BIC) stands for Akaike (Schwarz-Bayes) Information Criterion (25, 33), CFI stands for comparative fit index, CI stands for confidence interval, RMSEA is the root mean square error of approximation and SRMR is the standard root mean square residual.

## 4. Discussion

We report here the results of an investigation on latent variable structures of health-related responses from the intake survey users of the NURO app by means of three statistical and machine learning tools, namely variational autoencoders, exploratory factor analyses and confirmatory factor analyses. We primarily used the VAE for exploratory purposes to determine plausible lower dimensional latent structures behind the survey data. The VAE results of Figure 1 provided a strong indication that the data could be interpreted with a small number of latent variables or factors. The distribution of participants' responses on each of the 12 questions over the VAE latent space could be approximately classified into one of two types: one that aligns with the “Exercise-Diet” axis, and the other with the “Stress-Sleep” axis. The graphical results suggest that types of responses pertaining to exercise and diet exhibit correlations with each other, as do responses pertaining to stress and sleep, while other pairings of response types (e.g., stress and exercise) appear to have only minimal correlations. To provide additional contexts to the VAE results and also to ensure correct specification of subsequent CFA models, we also performed exploratory factor analyses (EFAs) on the same subset of data used for VAE training, the EFA results (Table 4) were in broad agreement with VAE observations.

Confirmatory factor analyses quantify the above observations. In each of the two-factor (Figure 2), three-factor (Figure 3) and four-factor (Figures 4, 5) CFA models developed, the fitted covariance parameters between the factors quantified the initial VAE results. In both the four-factor models (Figures 4, 5) for example, the fitted model covariance parameters were high between factors representing “exercise” and “diet”, and between factors representing “stress” and “sleep”, yet other fitted covariance parameter values between factors were comparatively much smaller or in some cases even statistically insignificant, effectively replicating the covariance structure of responses as observed in the latent space of the VAE.

An obvious advantage of our results is that the reduction in the apparent complexity of the response data paves the way for a more efficient stratification of participants based on the lower dimensional latent variables. This may allow better population selection for clinical trials and clustering of best drug efficacy discovery. Instead of considering the entire set of 12 response answers, a sufficient (and necessary) minimum of 2 latent variables account for a large portion of variation of responses of each participant. Moreover, the latent variables have a well-defined relationship with the original variables (i.e., “Exercise-Diet” and “Stress-Sleep”), providing immediate and reliable interpretation for clinicians and researchers alike utilizing these latent variables. Work is currently underway to investigate various performance metrics of participants also collected through the app and their association with the latent variables that are developed here.

Interestingly, in all of the VAE, EFA, and CFA results, there was an apparent anomaly that the responses of Q9 (“gastrointestinal issues”) aligned better with “Stress-Sleep” instead of “Exercise-Diet”. A plausible reason for the apparent anomaly could be the well-established link between the central and enteric nervous system, emotional and cognitive centers of the brain, and peripheral intestinal function *via* the gut-brain axis. This bidirectional system communicates from the gut-microbiota to the brain and from the brain to the gut-microbiota by means of neural, endocrine, immune, and humoral links (37). One last point on the anomaly is that the responses of the five questions aligning with the “Exercise-Diet” axis are all considered as “choices” and can be directly controlled by volitional actions of the user. Meanwhile, responses of Q9 pertaining to gastrointestinal issues after eating is more of a sensory input, as are the responses of other questions that align with “Stress-Sleep” axis. These are not directly controlled by one's volition. More research is necessary to explore this apparent anomaly which, on the other hand, could lead to interesting novel hypothesis on sleep rather than diet effects on intestinal health.

## Data availability statement

Supplementary material contain the raw data of all participants, in csv format, used for analyses of this work.

## Ethics statement

Ethical review and approval was not required for the study on human participants in accordance with the local legislation and institutional requirements. Written informed consent from the participants' legal guardian/next of kin was not required to participate in this study in accordance with the national legislation and the institutional requirements.

## Author contributions

DG and EH designed the study and wrote the first draft of the manuscript. DG developed the survey questionnaire. EH performed the machine learning and statistical analyses, which were reviewed by JG. JL led the development team of NURO app. DG, EH, JG, and LP wrote sections of the manuscript. All authors contributed to conception of the study, contributed to manuscript revision, read, and approved the submitted version.

## Conflict of interest

Authors DG, EH, JG, and JL are existing shareholders of Nurosene Health Inc. Author DG is the Co-founder of the company. Author JG is the Chief AI Scientist and Board Member at NetraMark Corp., which is a wholly owned subsidiary of Nurosene Health Inc., and they have worked with several pharmaceutical companies, including Takeda, INmune Bio, and Biohaven. Author LP is a medical advisor to Nurosene Health Inc., and for the past 2 years has consulted scientifically for Biogen, USA; Boehringer Ingelheim International GmbH, Germany; Compass Pathways, UK; EDRA-LSWR Publishing Company, Italy; Inpeco SA, Switzerland; Johnson and Johnson USA; Novartis and Avexis-Gene Therapies, Switzerland; Sanofi-Aventis-Genzyme, France and USA; Relmada Therapeutics, USA; WCG-Clinical Endpoint Solutions, USA.

## Publisher's note

All claims expressed in this article are solely those of the authors and do not necessarily represent those of their affiliated organizations, or those of the publisher, the editors and the reviewers. Any product that may be evaluated in this article, or claim that may be made by its manufacturer, is not guaranteed or endorsed by the publisher.
